# Soft selective sweeps: Addressing new definitions, evaluating competing models, and interpreting empirical outliers

**DOI:** 10.1371/journal.pgen.1010022

**Published:** 2022-02-24

**Authors:** Parul Johri, Wolfgang Stephan, Jeffrey D. Jensen

**Affiliations:** 1 School of Life Sciences, Arizona State University, Tempe, Arizona, United States of America; 2 Natural History Museum, Berlin, Germany; University of Rochester, UNITED STATES

## Abstract

The ability to accurately identify and quantify genetic signatures associated with soft selective sweeps based on patterns of nucleotide variation has remained controversial. We here provide counter viewpoints to recent publications in *PLOS Genetics* that have argued not only for the statistical identifiability of soft selective sweeps, but also for their pervasive evolutionary role in both *Drosophila* and HIV populations. We present evidence that these claims owe to a lack of consideration of competing evolutionary models, unjustified interpretations of empirical outliers, as well as to new definitions of the processes themselves. Our results highlight the dangers of fitting evolutionary models based on hypothesized and episodic processes without properly first considering common processes and, more generally, of the tendency in certain research areas to view pervasive positive selection as a foregone conclusion.

*“We would not object so strenuously to the adaptationist programme if its invocation, in any particular case, could lead in principle to its rejection for want of evidence. We might still view it as restrictive and object to its status as an argument of first choice. But if it could be dismissed after failing some explicit test, then alternatives would get their chance. Unfortunately, a common procedure among evolutionists does not allow such definable rejection for two reasons. First, the rejection of one adaptive story usually leads to its replacement by another, rather than to a suspicion that a different kind of explanation might be required. Secondly, the criteria for acceptance of a story are so loose that many pass without proper confirmation. Often, evolutionists use consistency with natural selection as the sole criterion and consider their work done when they concoct a plausible story.”*

- Gould and Lewontin, 1979 [1]

## Introduction

We write to provide counter viewpoints to those expressed by Garud, Messer, & Petrov 2021 [2] and Feder, Pennings, & Petrov 2021 [3], both of which were written in response to the work of Harris, Sackman, & Jensen 2018 [4].

There are 3 key points underlying this debate. The most specific of the 3 is in fact the most discussed, namely, the likely patterns of variation around a directly selected site upon the conclusion of an episodic bout of positive selection. The effect on linked sites related to this beneficial replacement is known as a selective sweep [5], and the details of expected patterns of genetic hitchhiking have been well reviewed in the literature [6–7]. In brief, a hard sweep refers to the subset of scenarios in which the beneficial variant exists on a single haplotype at the target of selection upon the conclusion of the sweep and is generally associated with a model in which the selective phase was entered while the beneficial allele was rare in the population. A soft sweep generally refers to a subset of scenarios in which multiple haplotypes carry the beneficial variant upon the conclusion of the sweep and is often associated with models in which the beneficial allele was either introduced into the population rapidly via independent mutational events (arising on distinct haplotypes, such that they may be distinguished from one another) or in which the variant was segregating as a common mutation on multiple distinct haplotypes at the onset of selection.

Importantly, both of these soft sweep–associated models may result in a hard sweep, depending on multiple underlying parameters [8–9]. As such, the hard versus soft sweep debate can neither be accurately characterized as addressing the likelihood of selection on new versus standing variation, but rather on rare versus common variation, nor as the likelihood of multiple cosegregating beneficial alleles, but rather as the likelihood of one of those alleles ultimately sweeping to fixation. Further, it has been well understood for decades that in recombining organisms such as *Drosophila melanogaster*, multiple haplotypes are expected in regions flanking beneficial fixations under a hard sweep model [5]. Thus, the single- versus multiple-haplotype sweep definition pertains to a highly localized genomic region—namely, the portion of the beneficial haplotype that does not experience a mutation or recombination event during the sweep. This pattern is additionally limited to a narrow temporal window post-sweep, as subsequent mutation, recombination, and genetic drift will quickly erode these hitchhiking patterns [10]. Given this high degree of specificity, Harris, Sackman, & Jensen 2018 [4] evaluated recent claims of widespread soft sweeps and found that this outcome could not be well discerned from those arising from competing models of hard sweeps or neutral population histories in the *Drosophila* and HIV populations here under discussion.

### Addressing newly arising definitions of soft selective sweeps

**Fig 1:**
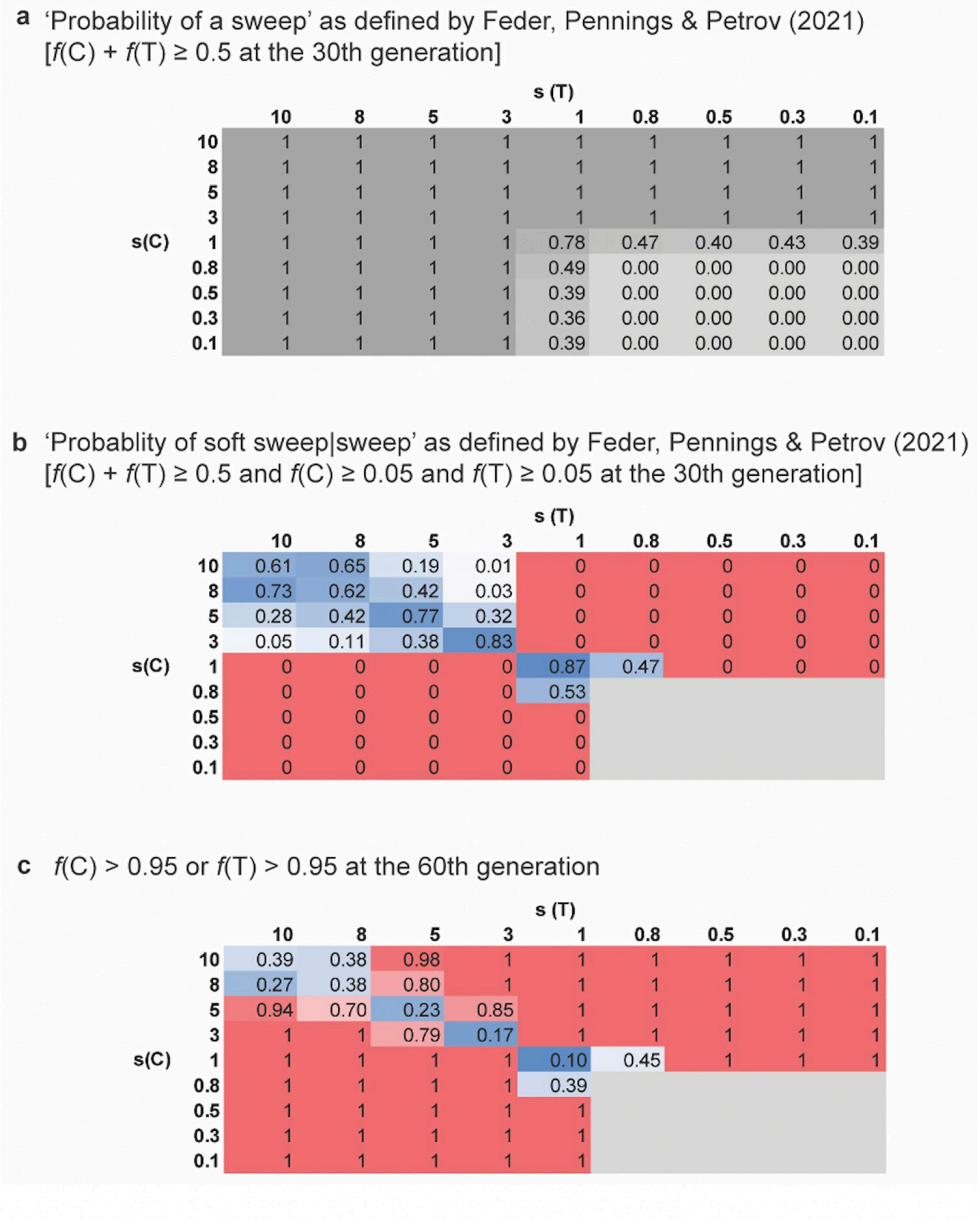
Frequencies of beneficial alleles C and T in the 30th and 60th generation post-onset of selection. The y-axis gives the selection coefficient of the beneficial mutation C, and the x-axis the selection coefficient of the beneficial mutation T. Thus, along the diagonals s(C) = s(T), whereas off the diagonals, there is a selective differential between the beneficial mutations. As shown in panel **(a)**, a beneficial mutation is likely to reach 50% frequency (their definition of a selective sweep) within 30 generations when selection coefficients are very strong. Panel **(b)** shows the proportions of replicates in which both beneficial nucleotides are at greater than 5% frequency given that the panel (a) condition has been met (their definition of a soft sweep). As shown, this is most likely when the selective effects are equal and falls off sharply with any selective differential. Finally, panel **(c)** follows the population to the 60th generation, demonstrating that an appreciable fraction of the scenarios meeting the ‘soft sweep’ definition of Feder, Pennings, & Petrov [3] in panel (b), in fact result in only a single nucleotide being brought to high frequency.

The second point underlying this debate concerns the model definitions themselves, as highlighted by the work of Feder, Pennings, & Petrov 2021 [3], who, in defending their earlier claims of soft sweeps in HIV [11], proposed definitions that are new to the field. Specifically, they define a ‘selective sweep’ as a single, or multiple, beneficial allele(s) at a site summing to 50% frequency in the population after 30 generations (as shown here in Fig 1A) and a ‘soft sweep’ as the subset of outcomes in which 2 of the alleles are present at 5% frequency or greater at that time (as shown in Fig 1B). These scenarios would commonly be instead referred to as a ‘partial selective sweep’ and as ‘co-segregating beneficial alleles,’ respectively. Importantly, for orientation, the conventional haplotype–based descriptions outlined above are not applicable in their model, as they consider only a single site. As such, they inherently invoke the multiple independent mutational event version of soft sweeps and utilize the presence of alternative nucleotides at the single site in question as a means of distinguishing soft sweeps (as determined by multiple common nucleotides) from hard sweeps (as determined by a single common nucleotide). Further, this work does not address the initial critiques of their Feder *et al.* [11] analysis (e.g., their assumptions that more effective and less effective treatments are characterized by the same infection history and selection coefficients) and represents a new line of argumentation.

These new definitions are quite different from the standard view of soft selective sweeps, as illustrated in their text: “*If 1 or more additional beneficial mutations enter the population before the first one fixes, the result is a soft sweep.*” That is, the assertion is that their definitions based on segregating allele frequencies at the single site in question are equivalent to the common definitions related to the conclusion of the sweep. However, this is not the case. Namely, while their criteria of a strongly selected beneficial mutation reaching 50% frequency is indeed highly predictive of a selective sweep [12], their criteria of 2 or more alleles being at 5% frequency or greater at that time is poorly predictive of a soft sweep. As shown in Fig 1C, when followed through time, a subset of replicates with identical selective effects that were identified by Feder, Pennings, & Petrov [3] as soft selective sweeps result in the fixation of a single nucleotide (as shown on the diagonal). It is also worth mentioning that the extreme selective effects that they considered (e.g., *s* = 1 or 5.2, corresponding to *Ns* values up to 5.2 × 10^6^) are in need of experimental validation.

More importantly, their single-site model neglects the fact that beneficial mutations occur on a genetic background, which is virtually guaranteed in HIV to contain other fitness-impacting variants given the high mutation rate. While there is some input of beneficial alleles, there is always a much higher input of deleterious variants in any functional region. Linkage with these variants will reduce the probability of beneficial fixation [13–17], and these background selection effects [18] have been examined in some depth [19–20]. Importantly, this genetic background may also serve to create selective differentials, even for beneficial mutations of identical effect arising at a single site. Specifically, if the deleterious loads on the genetic backgrounds differ, the probability of a soft selective sweep may be reduced, owing to the resulting fitness differential between haplotypes ([9]; and see [20]). The divergence in their model definitions relative to those commonly used by the field becomes similarly acute when one relaxes their assumption of all beneficial mutations being of equal effect size; namely their soft sweep definition may frequently be met (off the diagonal in Fig 1B), without resulting in a soft sweep at the time of fixation (off the diagonal in Fig 1C). Specifically, owing to the high mutation rates and strong selective pressures, multiple mutations may segregate at appreciable frequency; however, owing to competition between beneficials, the fittest nucleotide will likely experience a hard selective sweep, outcompeting the alternative nucleotides in relatively short order ([21–23]; and see [24–26]). This corresponding reduction in the probability of beneficial fixation has also been examined [27–29].

We also evaluated the simulation results of Feder, Pennings, & Petrov [3] analytically based on the classical selective sweep model of Maynard Smith & Haigh [5], who analyzed a 2-locus, 2-allele model of an infinitely large population. Such a deterministic model is appropriate when selection is very strong, as Feder, Pennings, & Petrov assume, and the frequency trajectory of the beneficial allele avoids the boundaries at 0 and 1. Kaplan *et al.* [12] and Stephan *et al.* [30] further considered a finite population size model and found that a strongly beneficial allele (with selection coefficient *s*) goes to fixation with high probability in a nearly deterministic fashion after its frequency in the initial phase (near boundary 0) has reached a threshold value of $x_{0}=\frac{5}{\alpha }$, where *α* = *Ns* (2*Ns*) in a haploid (diploid) population of size *N*. This prediction can be checked in Fig 3C of Feder, Pennings, & Petrov [3]. For example, for the upper curve (with *s* = 0.52), we find that the probability of a sweep is greater than 90% if *θ* = 1, which corresponds to *N* = 10^5^. Similarly, for the lower curve (*s* = 5.2) we find *P*(*sweep*)>90% for *θ* = 0.1, i.e., *N* = 10^4^. Note that the values of *Ns* are identical for these 2 examples, and in both cases, sweeps are predicted with high probability. However, as also shown in their Fig 3C, the probability of observing a soft sweep differs strongly between these 2 examples, owing partly to the poor predictability of their definition.

Importantly, in their considered parameter range of high *θ* values, their simulations also reveal that the perceived occurrence of ‘soft sweeps’ is no longer controlled by selection alone but by recurrent mutation as well. To further study this pattern, we introduced mutation from the wild type (WT) to the beneficial allele into the selective sweep model of Maynard Smith & Haigh [5]. The frequency change Δ*x* (per generation) of the beneficial allele during the fixation process under mutation and selection is then given as (see [31], Ch. 5)

Δx=μ(1−x)+sx(1−x)=(μ+sx)(1−x),

where *x* is the frequency of the beneficial allele, and *μ* is the mutation rate. Back mutation is neglected as in the stochastic initial phase *x* is very small, and the duration of the deterministic phase for $x\geq x_{0}=\frac{5}{Ns}$ is very short. For the high *θ* chosen in their Fig 3C, the mutational input (leading to soft sweeps) at frequencies around *x* = *x*_0_ (where the deterministic increase of the beneficial allele begins) is in the range of the selection pressure or even exceeding it. Indeed, for *θ* = 10, mutation is more important than selection such that the change in *x* is primarily owing to recurrent mutation: $\mu >sx_{0}=\frac{5}{N}$ (see equation above). In other words, in this parameter space, they are attributing mutation-driven frequency change to selection-driven frequency change (i.e., confounding mutational pressure with selection).

Hence, the dispute in question here neither concerns the fact that resistance evolution occurs in HIV populations, nor that multiple beneficial mutations may arise *de novo* in these patient populations given the high population mutation rates characterizing this virus. As previously pointed out by Jensen [9], if soft selective sweeps were to be observed in nature, such RNA viruses would be the likely candidates. However, as shown, a significant proportion of outcomes under the restrictive scenario considered by Feder, Pennings, & Petrov 2021 [3] actually result in only a single nucleotide being brought toward fixation. Furthermore, by relaxing their assumption of identical selective effects in a step toward biological reality, multi-nucleotide sweeps become increasingly unlikely, as would be expected. Finally, for much of the extreme parameter space chosen by the authors—in which *θ* is very large—mutation is more important than selection (i.e., the mutation rate is sufficiently high to itself contribute to observed allele frequency changes), suggesting caution when relying solely on selective sweep-based explanations.

### Fitting a baseline model, evaluating competing models, and interpreting empirical outliers

The third and much more general point fundamentally underlying this debate is well emphasized in the response of Garud, Messer, & Petrov 2021 [2], who defended their earlier claims of abundant soft sweeps in *D*. *melanogaster* [32] and relates to the interpretation of multiple competing models and empirical distributions. From their Abstract, “*Recently, Harris et al. 2018 criticized this work, suggesting that all the candidate loci detected by our haplotype statistics, including the positive controls, are unlikely to be sweeps at all and that instead these haplotype patterns can be more easily explained by complex neutral demographic models. They also claim that these neutral non-sweeps are likely to be hard instead of soft sweeps.*”

Of course, ‘hard neutral non-sweeps’ is a concept devoid of meaning, and their summary of this point instead reflects the demonstration of Harris, Sackman, & Jensen 2018 [4] that models of recurrent soft sweeps, recurrent hard sweeps, as well as a neutral non-equilibrium demographic histories, were all found to be consistent with the observed data. Garud, Messer, & Petrov suggest that this is tantamount to arguing that all of the models are true. However, this result rather demonstrates that the data are not sufficient to distinguish among these possibilities; thus, it is not possible to draw the strong conclusion that one of the competing models, such as recurrent soft sweeps, is the most likely. Nonetheless, Garud, Messer, & Petrov argue that the recurrent soft sweep model is correct [2], as their empirically observed outliers fall near the mean of the expected distribution of their proposed haplotype-based statistics under models of soft sweeps, whereas they fall in the tails of the distribution of both hard sweep and neutral demographic models (as shown in Fig 1 of [4]). While the generality of the soft sweep model in fact makes virtually any observation reasonably likely for the chosen haplotype statistics of Garud *et al.* 2015 [32], their analysis was based on a scan of the Drosophila Genetic Reference Panel (DGRP) data, and their candidate loci were identified as being outliers in the empirical genomic distribution. Thus, it is unclear why the authors feel that their observed empirical outliers would be inconsistent with alternative models that predict such outlier values.

More importantly, however, this speaks to the general notion of fitting an appropriate null model. By their own admission, the authors neither fit nor identify a null model that actually predicts the general features of the data. For example, they note that the recently published Arguello *et al.* model [33] does not predict general levels or patterns of empirical diversity. However, this is not particularly surprising, given that the Arguello *et al.* model was fit to an Ithaca, New York population (using putatively neutral sites), while Garud, Messer, & Petrov are instead considering the DGRP data (including coding regions, in which selective sweeps are hypothesized to be occurring). Rather than fitting an appropriate null for their studied population, they instead simulate a variety of models and conclude that they have not identified a model that is predictive of their 5 basic summary statistics considered. With this result, they suggest that because accurate demographic model fitting is difficult, one may simply focus on “*extreme outliers of the scan, as these candidates are the least likely to be sensitive to the choice of demographic model.*” While the justification for this statement is unclear, this is not advisable. First, any distribution has tails. Under a neutral model, if one takes 5% or 1% of outliers of a given statistic as meaningful, one will incorrectly identify 5% or 1% of the loci as having experienced a sweep. On the other side of the coin, under non-equilibrium models with selection, it is not a given that positively selected loci would be expected to occupy the tails of a given statistical distribution [34]. Thus, this ‘empirical outlier’ approach—advocated in their Box 1 and throughout as an alternative to careful model fitting—may be characterized by extreme false-positive rates and/or low power, to an extent that may in fact itself only be determined via careful model fitting. Moreover, as the DGRP population is highly bottlenecked, this type of approach is particularly problematic [35].

As a simple example for the purposes of clarification, imagine sequencing a hypothetical population and finding that the mean genome-wide value of Tajima’s *D* is −0.2 and that the most extreme observed negative value at any locus is −1.0. This alone is simply a descriptive result of the data and in the absence of population genetic modeling does not tell us about acting evolutionary processes shaping this observed distribution. That is, there is no reason to conclude based on this information alone that the most negative locus is the result of a selective sweep, simply because selective sweeps may generate negative values of Tajima’s *D*; that may indeed be a hypothesis of interest, but one would first need to model the details of the population history (i.e., as population size change may also generate negative values), functional constraint (i.e., as purifying and background selection may also generate negative values), and so on, in order to determine if this observed tail is expected under such a basic model, and if not, to next determine whether violations of that null model (including positive selection) would be likely to produce this observation within the context of the fit model. In the absence of that exercise, there is no justification for assigning any evolutionary process as a driver of this ‘outlier.’ To belabor the parallel with the discussed Garud, Messer, & Petrov study, if rather than fitting a null model one instead arbitrarily assigned a model as the null that had a mean Tajima’s *D* of 0.5 (rather than the empirically observed mean of −0.2), and based on that comparison determined that the empirical outlier is indeed the result of positive selection because it appears to be extreme under the assigned ‘null,’ that too would be inappropriate as there would be no basis for studying the tail of the distribution when the entirety of the distribution was itself unlike the observed data.

**Fig 2:**
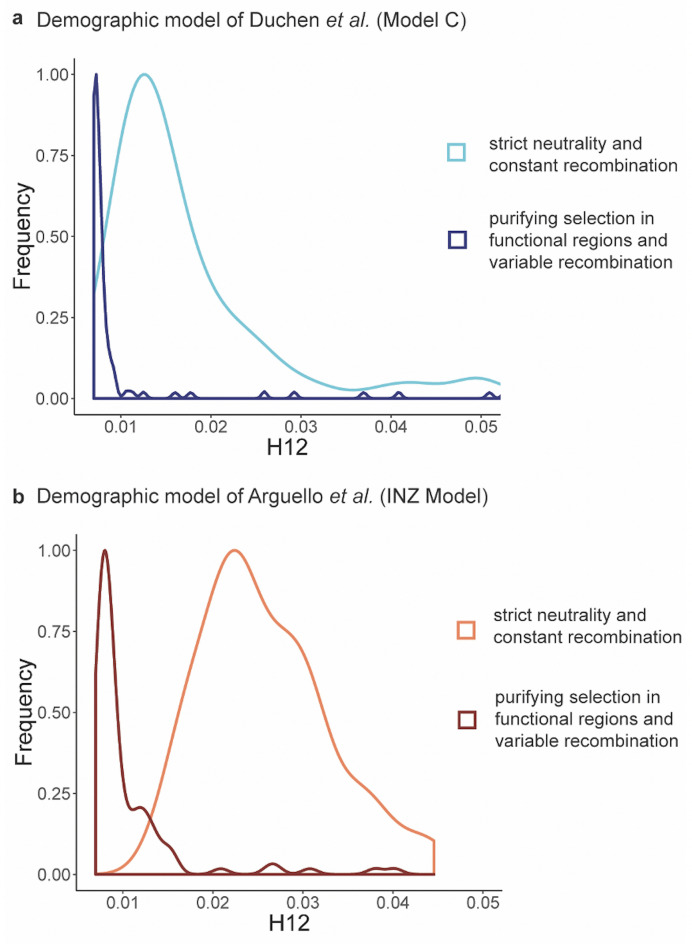
The distributions of the H12 statistic (from 100-kb regions, calculated in 401 SNP sliding windows following [2]) in the simulated Raleigh and Ithaca populations under the slightly modified best-fit demographic models inferred by **(a)** Duchen *et al.* 2013 [40] and **(b)** Arguello *et al.* 2019 [33]. For each demographic model, 2 scenarios are shown: strict neutrality with a constant recombination rate of 0.5 cM/Mb, as assumed by Garud, Messer, & Petrov [2] (100 replicates) and purifying selection in functional regions using the DFE of deleterious mutations inferred by Johri *et al.* 2020 [38], with variable recombination rates sampled from the *D*. *melanogaster* genome (100 replicates). The x-axes are truncated at 0.05 to show a clearer visualization of the means of the distributions. DFE, distribution of fitness effects.

Thus, in order to better quantify some of the consequences associated with their lack of an appropriate null model, we evaluated 2 of their assumptions for the sake of example. First, the authors assume a constant recombination rate across the genome, despite substantial evidence of rate heterogeneity [36–37]. Second, they neglect the effects of purifying selection, which have been argued to be extensive in *D*. *melanogaster* [38–39]. As recombination rate heterogeneity, purifying selection, and background selection would be expected to impact the scale and variance of haplotype distributions, and as Garud, Messer, & Petrov argue that a strictly neutral demographic model does not explain the empirical distribution of their statistic under their given assumptions, these processes require investigation as they would represent essential components of an appropriate null model. As shown in Fig 2, the inclusion of realistic purifying selection acting on functional regions, as well as a *Drosophila*-like recombination rate map, strongly shape the expected H12 distributions relative to the strictly neutral and constant rate demographic models of Arguello *et al.* [33] and Duchen *et al.* [40] used by Garud, Messer, & Petrov. Notably, the inclusion of these more realistic considerations also left shifts the means of these null distributions to be very similar to that observed empirically in the DGRP data and thereby strongly suggests the need for fitting a correct null model. Hence, attributing deviations to positive selection, while neglecting these known processes that are strongly shaping the empirical distribution, is inherently problematic. If, upon fitting such a null model, there remain tails of empirical distributions that are not explainable by the underlying variance related to these common evolutionary processes, one may then begin evaluating hypothesized episodic processes (e.g., selective sweeps, among any other likely null model violations) within the context of this fit model in order to firstly determine if these additional processes are statistically identifiable and, if so, whether they represent viable model additions for better explaining the full empirical distribution.

With a properly fit null model, the ultimate question of interest becomes how to interpret the fit of multiple opposing models. The belief that the correlation between recombination rates and levels of variation was generated by recurrent sweeps [41] was tempered by the finding that linkage to deleterious rather than beneficial mutations may also produce this relationship [18]; the belief that Fay and Wu’s *H*-statistic demonstrated genome-wide sweeps [42] was tempered by the finding that neutral demographic models may result in similar statistical distributions [10]. Importantly, the observation that genetic drift or background selection are viable alternatives is not equivalent to claiming that recurrent sweep models are irrelevant or incorrect; similarly, the finding that neutral null models or hard sweeps may account for patterns attributed to soft sweeps is not itself evidence against soft sweeps. It is, however, an important call for caution against overinterpreting data and against promoting specific models without considering realistic alternatives. The observation that opposing models may be fit to a given data observation is a statement of identifiability—that is, additional data and/or analysis is needed to better distinguish the possibilities.

## Conclusions

In evolutionary genomic analyses, there exist compelling reasons to begin with a null baseline model consisting of evolutionary processes that are certain to be in constant operation, as opposed to models centered upon hypothesized and episodic processes such as selective sweeps [43–45]. In both of the examples discussed here, such a model will include the genetic drift inherent to all finite populations as modulated by population history, the purifying and background selection effects resulting from the constant input of deleterious alleles in functional genomic regions, as well as realistic mutation and recombination rate parameters. For the *D*. *melanogaster* data reanalyzed by Garud, Messer, & Petrov 2021 [2], their lack of an appropriate null model, combined with their reliance on empirical outliers as an alternative, continues to cast considerable doubt on their claims of pervasive soft sweeps. For the HIV data reanalyzed by Feder, Pennings, & Petrov 2021 [3], the lack of consideration of linked and variable selective effects and mutation-driven allele frequency change, along with the use of uncommon definitions, has similarly led to a biased emphasis on soft sweep outcomes.

## Methods

### Simulations of sweeps in HIV-like populations

All simulations were performed in SLiM 3.3.1 [46]. For every parameter combination, 100 replicates were simulated. A diploid population was simulated with a constant size of 10^5^ and a constant mutation rate (*μ*) of 10^−5^ per site per replication. A single site was simulated with a finite-site nucleotide model (with an equal probability of mutation to each nucleotide). For the site in question, the allele “*A*” was the WT state, “*G*” was WT equivalent, while “*C*” and “*T*” both represented beneficial mutations with selective effects *s*(*C*) and *s*(*T*), respectively. All mutations were semidominant with the following genotype fitness: *w*_*AA*_ = *w*_*GG*_ = *w*_*AG*_ = 1; *w*_*TT*_ = 1+*s*(*T*); *w*_*CC*_ = 1+*s*(*C*); *w*_*AT*_ = *w*_*GT*_ = 1+0.5*s*(*T*); *w*_*AC*_ = *w*_*GC*_ = 1+0.5*s*(*C*); *w*_*TC*_ = 1+max (*s*(*C*), *s*(*T*)). The forward simulations began with the site fixed for the WT allele “*A*” and were evaluated at 30 and 60 generations, likely corresponding to the first few months of treatment given an estimated generation time of 1 to 2 days [47–48]. Scripts used to perform these simulations can be found at https://github.com/paruljohri/PlosGenViewpoint/tree/main/HIV.

### Simulations of *D*. *melanogaster* populations

All simulations were performed for a 100-kb genomic element. For constant recombination rates, 0.5cM/Mb was used to match simulations performed by Garud, Messer, & Petrov 2021 [2]. To model variable recombination rates, rates were calculated for 10-kb regions across the chromosomes 2 and 3, with rates of recombination as specified by Comeron *et al.* 2012 [36]. Random samples of 100-kb regions (i.e., 10 contiguous 10-kb regions) were sampled with replacement from the *D*. *melanogaster* genome. Following Garud, Messer, & Petrov, regions with rates smaller than 0.5cM/Mb were excluded (i.e., 100kb elements were resampled until regions were obtained with rates greater than 0.5cM/Mb). The same recombination rates were used when simulating the 2 demographic models.

When simulating purifying selection, 33 genes were simulated within the 100-kb region, such that each gene was comprised of 5 exons (of 300 bp) and 4 introns (of 100 bp). Intergenic regions were comprised of 1,068 bp, such that exons represent 49.5% of the genome. Exonic sites experienced purifying selection specified by the distribution of fitness effects (DFE) of new deleterious mutations estimated by Johri *et al.* 2020 [38]. Specifically, 23% of all new mutations were strictly neutral, 51% were mildly deleterious with −10≤2*N*_*anc*_*s*<−1, 4% were moderately deleterious with −100≤2*N*_*anc*_*s*<−10, and 22% were strongly deleterious such that 2*N*_*anc*_*s*<−100. For these intervals, selection against homozygotes (*s*) was sampled uniformly, and all mutations were assumed to be semidominant. *N*_*anc*_ here refers to the ancestral population size, which was the ancestral size of the Zimbabwe population in both demographic models.

Simulations of the North American populations of *D*. *melanogaster* were simulated under 2 different demographic models: (1) a slightly modified version of the best-fit model inferred by Duchen *et al.* 2013 [40] (Model C), where the Raleigh population was assumed to be of constant size (equal to the current size of the population) post-admixture, and the mutation rate was 1.0×10^−9^ per site/generation, and 2) a slightly modified version of the best-fit model inferred by Arguello *et al.* 2019 [33] (INZ Model), where the Ithacan population was assumed to be of constant population size (equal to the current size of the population) post-admixture, and the mutation rate was 1.39×10^−9^ per site/generation. The mode of the posterior distributions of parameter values provided in both studies was used for simulations. Simulations were performed in SLiM 3.1 [46] where all parameters were rescaled by 100 and 300 for the Arguello *et al.* and Duchen *et al.* models, respectively, and 100 replicates were simulated for each. The population sizes, timing, and duration of events were scaled down, while mutation, recombination, and migration rates were scaled up by the same factor. Mean [SD] values of the H12 statistic under strict neutrality and with a recombination rate of 0.5 cM/Mb obtained using the rescaled models in SLiM: 0.0261 [0.0082] and 0.0216 [0.0121]; matched the values obtained from simulations performed with no rescaling in msprime 0.7.3 [49]: 0.0270 [0.0057] and 0.0233 [0.0151], for the demographic models of Arguello *et al.* and Duchen *et al.*, respectively. The slightly modified versions of the demographic models were simulated because the number of generations required for growth in the Ithacan and Raleigh populations were extremely small under the rescaled models.

Samples of 145 genomes were recorded from each of the North American populations—Raleigh and Ithaca. H12 stats were calculated using the script provided by Garud, Messer, & Petrov [2]. All scripts used to perform simulations and to calculate statistics can be found at https://github.com/paruljohri/PlosGenViewpoint/tree/main/Drosophila.

## References

[1] Gould SJ, Lewontin RC. The spandrels of San Marco and the Panglossian paradigm: a critique of the adaptationist programme. Proc R Soc Lond B. 1979;205:581–98. doi: 10.1098/rspb.1979.0086 42062

[2] Garud N, Messer P, Petrov D. Detection of hard and soft selective sweeps from Drosophila melanogaster population genomic data. PLoS Genet. 2021;17:e1009373. doi: 10.1371/journal.pgen.1009373 33635910 PMC7946363

[3] Feder AF, Pennings PS, Petrov DA. The clarifying role of time series data in the population genetics of HIV. PLoS Genet. 2021;17:e1009050. doi: 10.1371/journal.pgen.1009050 33444376 PMC7808693

[4] Harris RB, Sackman A, Jensen JD. On the unfounded enthusiasm for soft selective sweeps II: examining recent evidence from humans, flies, and viruses. PLoS Genet. 2018;14(12):e1007859. doi: 10.1371/journal.pgen.1007859 30592709 PMC6336318

[5] Maynard Smith J, Haigh J. The hitch-hiking effect of a favourable gene. Genet Res. 1974;23:23–5. 4407212

[6] Stephan W. Selective sweeps. Genetics. 2019;211:5–13. doi: 10.1534/genetics.118.301319 30626638 PMC6325696

[7] Charlesworth B, Jensen JD. The effects of selection at linked sites on patterns of genetic variability. Annu Rev Ecol Evol Syst. 2021;52:177–97. 10.1146/annurev-ecolsys-010621-044528 PMC10120885 37089401

[8] Orr HA, Betancourt AJ. Haldane’s sieve and adaptation from standing genetic variation. Genetics. 2001;157:875–84. doi: 10.1093/genetics/157.2.875 11157004 PMC1461537

[9] Jensen JD. On the unfounded enthusiasm for soft selective sweeps. Nat Commun. 2014;5:5281. doi: 10.1038/ncomms6281 25345443

[10] Przeworski M. The signature of positive selection at randomly chosen loci. Genetics. 2002;160:1179–89. doi: 10.1093/genetics/160.3.1179 11901132 PMC1462030

[11] Feder AF, Rhee S-Y, Holmes SP, Shafer RW, Petrov DA, Pennings PS. More effective drugs lead to harder selective sweeps in the evolution of drug resistance in HIV-1. Elife. 2016;5:e10670. doi: 10.7554/eLife.10670 26882502 PMC4764592

[12] Kaplan NL, Hudson RR, Langley CH. The ‘hitchhiking effect’ revisited. Genetics. 1989;123:887–99. doi: 10.1093/genetics/123.4.887 2612899 PMC1203897

[13] Charlesworth B. The effect of background selection against deleterious mutations on weakly selected, linked variants. Genet Res. 1994;63:213–27. doi: 10.1017/s0016672300032365 8082838

[14] Charlesworth B. The effects of deleterious mutations on evolution at linked sites. Genetics. 2012;190:5–22. doi: 10.1534/genetics.111.134288 22219506 PMC3249359

[15] Stephan W, Charlesworth B, McVean G. The effects of background selection at a single locus on weakly selected, partially linked variants. Genet Res. 1999;73:133–46.

[16] Rouzine I, Wakeley J, Coffin JM. The solitary wave of asexual evolution. Proc Natl Acad Sci U S A. 2003;100:587–92. doi: 10.1073/pnas.242719299 12525686 PMC141040

[17] Jain K. Interference effects of deleterious and beneficial mutations in large asexual populations. Genetics. 2019;211:1357–69. doi: 10.1534/genetics.119.301960 30700529 PMC6456326

[18] Charlesworth B, Morgan MT, Charlesworth D. The effect of deleterious mutations on neutral molecular variation. Genetics. 1993;134:1289–303. doi: 10.1093/genetics/134.4.1289 8375663 PMC1205596

[19] Weissmann DB, Barton NH. Limits to the rate of adaptive substitution in sexual populations. PLoS Genet. 2012;8:e1002740. doi: 10.1371/journal.pgen.1002740 22685419 PMC3369949

[20] Pennison S, Singh T, Sniegowski P, Gerrish P. Dynamics and fate of beneficial mutations under lineage contamination by linked deleterious mutations. Genetics. 2017;205:1305–18. doi: 10.1534/genetics.116.194597 28100591 PMC5340340

[21] Fisher RA. The genetical theory of natural selection. 1930; Oxford, UK: Clarendon Press.

[22] Muller HJ. Some genetic aspects of sex. Am Nat. 1932;66:118–38.

[23] Hill WG, Robertson A. The effect of linkage on limits to artificial selection. Genet Res. 1966;8:269–94. 5980116

[24] Barton NH. Linkage and the limits to natural selection. Genetics. 1995;140:821–44. doi: 10.1093/genetics/140.2.821 7498757 PMC1206655

[25] Kim Y, Stephan W. Selective sweeps in the presence of interference among partially linked loci. Genetics. 2003;164:389–98. doi: 10.1093/genetics/164.1.389 12750349 PMC1462541

[26] Desai MM, Fisher DS. Beneficial mutation selection balance and the effect of linkage on positive selection. Genetics. 2007;176:1759–98. doi: 10.1534/genetics.106.067678 17483432 PMC1931526

[27] Gerrish PJ, Lenski RE. The fate of competing beneficial mutations in an asexual population. Genetica. 1998;102(103):127–44. 9720276

[28] Sniegowski PD, Gerrish PJ. Beneficial mutations and the dynamics of adaptation in asexual populations. Phil Trans R Soc B. 2010;365:1255–63. doi: 10.1098/rstb.2009.0290 20308101 PMC2871819

[29] Kim Y, Orr HA. Adaptation in sexuals vs. asexuals: clonal interference and the Fisher–Muller model. Genetics 2005; 171: 1377–86. doi: 10.1534/genetics.105.045252 16020775 PMC1456838

[30] Stephan W, Wiehe THE, Lenz MW. The effect of strongly selected substitutions on neutral polymorphism: analytical results based on diffusion theory. Theoret Popul Biol. 1992;41:237–54.

[31] Ewens WJ. Mathematical population genetics: I. Theoretical introduction, 2nd ed. Springer. 2004.

[32] Garud N, Messer P, Buzbas E, Petrov D. Recent selective sweeps in North American Drosophila melanogaster show signatures of soft sweeps. PLoS Genet. 2015;11:e1005004. doi: 10.1371/journal.pgen.1005004 25706129 PMC4338236

[33] Arguello JR, Laurent S, Clark AG. Demographic history of the human commensal Drosophila melanogaster. Gen Biol Evol. 2019;11:844–54. doi: 10.1093/gbe/evz022 30715331 PMC6430986

[34] Teshima K, Coop G, Przeworski M. How reliable are empirical genome scans for selective sweeps? Genome Res. 2006;16:702–12. doi: 10.1101/gr.5105206 16687733 PMC1473181

[35] Thornton KR, Jensen JD. Controlling the false positive rate in multi-locus genome scans for selection. Genetics. 2007;175:737–50. doi: 10.1534/genetics.106.064642 17110489 PMC1800626

[36] Comeron JM, Ratnappan R, Bailin S. The many landscapes of recombination in Drosophila melanogaster. PLoS Genet. 2012;8:e1002905. doi: 10.1371/journal.pgen.1002905 23071443 PMC3469467

[37] Hunter CM, Huang W, Mackay TFC, Singh ND. The genetic architecture of recombination rate variation in Drosophila melanogaster. PLoS Genet. 2016;12:e1005951. doi: 10.1371/journal.pgen.1005951 27035832 PMC4817973

[38] Johri P, Charlesworth B, Jensen JD. Towards an evolutionarily appropriate null model: jointly inferring demography and purifying selection. Genetics. 2020;215:173–92. doi: 10.1534/genetics.119.303002 32152045 PMC7198275

[39] Johri P, Riall K, Becher H, Excoffier L, Charlesworth B, Jensen JD. The impact of purifying and background selection on the inference of population history: problems and prospects. Mol Biol Evol. 2021;38:2986–3003. doi: 10.1093/molbev/msab050 33591322 PMC8233493

[40] Duchen P, Zivkovic D, Hutter S, Stephan W, Laurent S. Demographic inference reveals African and European admixture in the North American Drosophila melanogaster population. Genetics. 2013;193:291–301. doi: 10.1534/genetics.112.145912 23150605 PMC3527251

[41] Begun DJ, Aquadro CF. Levels of naturally occurring DNA polymorphism correlated with recombination rates in D. melanogaster. Nature. 1992;356:519–20. doi: 10.1038/356519a0 1560824

[42] Fay J, Wu C-I. Hitchhiking under positive Darwinian selection. Genetics. 2000; 155: 1405–13. doi: 10.1093/genetics/155.3.1405 10880498 PMC1461156

[43] Comeron JM. Background selection as a baseline for nucleotide variation across the Drosophila genome. PLoS Genet. 2014;10(6):31004434. doi: 10.1371/journal.pgen.1004434 24968283 PMC4072542

[44] Pouyet F, Aeschbacher S, Thiery A, Excoffier L. Background selection and biased gene conversion affect more than 95% of the human genome and bias demographic inferences. Elife. 2018;7:e36317. doi: 10.7554/eLife.36317 30125248 PMC6177262

[45] Jensen JD, Payseur BA, Stephan W, Aquadro CF, Lynch M, Charlesworth D, et al. The importance of the Neutral Theory in 1968 and 50 years on: a response to Kern & Hahn 2018. Evolution. 2019;73:111–4. doi: 10.1111/evo.13650 30460993 PMC6496948

[46] Haller BC, Messer PW. SLiM 3: Forward genetic simulations beyond the Wright-Fisher model. Mol Biol Evol. 2019;36:632–7. doi: 10.1093/molbev/msy228 30517680 PMC6389312

[47] Rodrigo AG, Shpaer EG, Delwart EL, Iversen AKN, Gallo MV, Brojatsch J, et al. Coalescent estimates of HIV-1 generation time in vivo. Proc Natl Acad Sci U S A. 1999;96:2187–91. doi: 10.1073/pnas.96.5.2187 10051616 PMC26758

[48] Fu Y-X. Estimating mutation rate and generation time from longitudinal samples of DNA sequences. Mol Biol Evol. 2001;18:620–6. doi: 10.1093/oxfordjournals.molbev.a003842 11264414

[49] Kelleher J, Etheridge AM, McVean G. Efficient coalescent simulation and genealogical analysis for large sample sizes. PLoS Comput Biol. 2016;12:e1004842. doi: 10.1371/journal.pcbi.1004842 27145223 PMC4856371

